# Preclinical Models in Prostate Cancer: Resistance to AR Targeting Therapies in Prostate Cancer

**DOI:** 10.3390/cancers13040915

**Published:** 2021-02-22

**Authors:** Wout Devlies, Florian Handle, Gaëtan Devos, Steven Joniau, Frank Claessens

**Affiliations:** 1 Laboratory of Molecular Endocrinology, KU Leuven, 3000 Leuven, Belgium; wout.devlies@kuleuven.be; 2 Department of Urology, University Hospitals Leuven, 3000 Leuven, Belgium; gaetan.devos@uzleuven.be (G.D.); steven.joniau@uzleuven.be (S.J.); 3 Division of Experimental Urology, Department of Urology, Medical University of Innsbruck, 6020 Innsbruck, Austria; florian.handle@i-med.ac.at

**Keywords:** preclinical models, androgen receptor, prostate cancer, hormone treatment, ARSI, ARTA, treatment resistance

## Abstract

**Simple Summary:**

In this review, we will look into the existing methods to study treatment resistance to androgen receptor targeted therapies in prostate cancer. This will encompass both the different existing preclinical models as well as the role specific models have played in the current understanding of resistance mechanisms.

**Abstract:**

Prostate cancer is an androgen-driven tumor. Different prostate cancer therapies consequently focus on blocking the androgen receptor pathway. Clinical studies reported tumor resistance mechanisms by reactivating and bypassing the androgen pathway. Preclinical models allowed the identification, confirmation, and thorough study of these pathways. This review looks into the current and future role of preclinical models to understand resistance to androgen receptor-targeted therapies. Increasing knowledge on this resistance will greatly improve insights into tumor pathophysiology and future treatment strategies in prostate cancer.

## 1. Introduction

**Figure 1:**
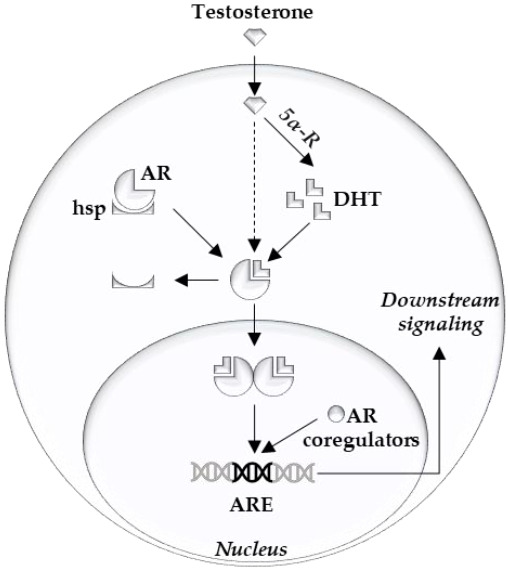
Overview of intracellular androgen action. Androgen receptor (AR), heat shock proteins (hsp), dihydrotestosterone (DHT), 5-alpha reductase (5α-R), androgen response element (ARE).

The androgen receptor (AR) is the main driver of proliferation in prostate cancer (PCa) cells, and thus is an important therapeutic target [1,2]. It consists of three structural components: the C terminal domain/ligand binding domain, the DNA binding domain, and the N terminal domain [3]. Ligand binding, dimerization, and complex formation with coregulators contribute to the downstream AR signaling [4] (Figure 1). At the cellular level, prostate epithelial cells depend on survival- and growth-inducing signals from the AR. Thus, AR inhibition deprives PCa cells of these signals and leads to G1 arrest [5].

Clinical targeting of the AR is achieved by LHRH agonists/antagonists and abiraterone or by blocking the AR ligand binding pocket with anti-androgens (e.g., apalutamide, darolutamide, and enzalutamide). Unfortunately, curative treatment of advanced PCa by these AR signaling inhibitors is prevented by the occurrence of resistance mechanisms [1,2].

To become resistant against these therapies, PCa cells have to overcome the lack of growth signals. The mechanisms can be broadly categorized into two groups: (1) mechanisms that reactivate AR signaling output (i.e., AR overexpression, mutation and splice variants, glucocorticoid receptor takeover, androgen synthesis); and (2) mechanisms that bypass the AR by providing growth signals via other means (i.e., alterations in cell cycle regulators, DNA repair pathways, PI3K/PTEN alterations, trans-differentiation into AR independent phenotypes such as neuroendocrine cells).

## 2. Overview of Preclinical PCa Models

### 2.1. Prostate Cancer Derived Cell Lines

#### 2.1.1. Cell Lines

Different cell types are used as preclinical PCa models, each with their distinct features. The most commonly used PCa cell lines and their characteristics are shown in Table 1.

LNCaP is a cell line derived from a lymph node metastasis. It harbors a T877A AR mutation, with ligand promiscuity and has only a modest potential to form xenografts in nude mice [9]. It has multiple derivative cell lines, differing from the maternal LNCaP cell lines on different points: C4-2b (PSA +, bone metastatic in mice), LNCaP-abl (androgen-resistant, androgen depleted medium), LNCaP-AR (androgen-resistant, tissue culture), ResA and B (androgen- and anti-androgen-resistant) [10,11]. VCaP cells come from a metastatic lesion and have a wild-type, yet amplified, AR. VCaP cells are especially interesting in preclinical research as they express the TMPRSS2-ERG gene arrangement, commonly seen in PCa patients [12]. DuCaP is derived from a hormone refractory xenograft of a dura mater metastasis and is expressing PSA and overexpressing wildtype AR [7,13]. Multiple LAPC cell lines are androgen-responsive and developed from a xenograft of a PCa lymph node (LAPC-4) and bone (LAPC-9) metastasis. LAPC-4 is characterized by mutations of p53 and wildtype PTEN [14]. DU-145 are derived from a PCa brain metastasis. It does not express AR and is therefore hormone-insensitive [15]. PC3 cells are grown from a PCa bone metastasis and are, similarly to DU-145, AR negative. [16]. Some studies suggested PC3 cells to have neuroendocrine features reflecting small cell PCa and stem-like features [17,18]. 22Rv1 is a cell line derived from a relapsing xenograft of the CWR22 cell line in mice that were castrated [19]. Its AR is mutated (H874Y), while expressing a truncated AR in parallel because of an intragenic duplication of exon 3 and has a wildtype PTEN. It is mostly used in research involving splice variants of the AR [8]. MDA PCa 2A is a cell line derived from bone metastasis, being AR positive, harboring AR mutants L702H and T878A [6,20].

The big advantages of these cell lines are their relative simplicity and ease of culturing in different conditions. This enables the rapid validations of hypotheses and the simultaneous testing of multiple compounds. Also beneficial is the high amount of data that are already available on them (i.e., on DepMap and CCLE) [21,22]. Each cell line should, however, be considered as a proxy of a very specific type of PCa (i.e., AR/PSA positive, TMPRSS2-ERG fusion positive, PTEN status) (Table 3).

For example, in Tran et all., they used LNCaP cells with increased expression of wild type AR and VCaP cells. In these cells, together with the derived xenograft model, the AR inhibitor MDV3100 was identified, which was later renamed to enzalutamide [23]. PCa cell lines play an important role in investigating the plethora of AR resistance mechanisms. Such resistance to AR signaling inhibitors may pre-exist in the patient (de novo resistance) or may develop upon treatment of the patient (acquired resistance). Both types of resistance can be recapitulated in culture conditions, which enables researchers to study them in detail.

De novo resistance mechanisms can be studied in cellular models generated via genetically modifying PCa cells (i.e., stable transfections, up/down-regulation of a gene by siRNA or CRISPR/Cas9), but this largely limits their use in validation studies. In contrast to de novo resistance studies, cell lines with acquired resistance may lead to the discovery of novel resistance mechanisms, but it is challenging to identify the underlying mechanism [11]. More recently, genome-wide CRISPR-Cas9 screenings allowed the discovery of novel resistance mechanisms [24]. While these cell lines certainly have their place in the preclinical research landscape, they do not reflect all aspects of the heterogeneous primary tumor biology. This makes investigating, for example, the role of tumor microenvironment difficult, as only one cell type is present in the cultures. Another shortcoming is that cell lines all have severe aneuploidy. For example, the complex chromosomes of LNCaP (87 chromosomes), PC3 (58/113), and DU145 (62 chromosomes) have structural alterations of multiple chromosomes (1, 2, 4, 6, 10, 15, and 16) [25].

#### 2.1.2. Xenografts and 3D models

Mouse xenografts are commonly created by transplanting human cell lines in mice. This can be performed in different locations: subrenal (under the renal capsule), orthotopic (in the mouse prostate), and subcutaneous (under the skin), each with its specific characteristics [8]. Creating these xenografts allows the analysis of metastasis and treatment response in an in vivo setting (Table 3).

For example, in Nguyen et al., multiple PDX were developed, reflecting prostate cancer features such as AR amplification, PTEN deletion, TP53 deletion/mutation, RB1 loss, TMPRSS2-ERG rearrangements, SPOP mutation, MSH2/MSH6 genomic aberrations, and BRCA2 loss [26]. From these models, Lam et al. selected the enzalutamide-resistant models to study supraphysiological testosterone treatment as a resensitization strategy for AR-targeted therapies [27].

Three-dimensional cell cultures can similarly be valuable in creating a basis to investigate cell–cell interactions. Cell lines can consequently be studied in a tumor microenvironment [8,28]. This can be made from artificial or biologic materials. The effects of the artificially engineered 3D scaffolds on the cells and their androgen sensitivity remain under investigation [28,29].

For example, in the spheroids of Eder et al., cancer-associated fibroblasts were co-cultured with LNCaP, LAPC4, and DuCaP. This made DuCaP resistant to enzalutamide, by increasing the Akt PI3K pathway (see further). Novel treatments can be proposed and tested in these resistant tumors [30].

### 2.2. Genetically-Engineered Mouse Models (GEMM)

Transgenic technology has enabled mimicking genetic changes that have been discovered in PCa. The genes under investigation can be knocked out or altered specifically in mouse prostate luminal cells. In this way, GEMMs of different pathways that lead to treatment resistance in humans have been recreated, of which the most commonly altered mechanisms are represented in Table 2 [31,32].

As a consequence, human PCa phenotypes can be mimicked in GEMMs, and murine metastatic signatures that can be relevant in humans can be discovered [33]. While inter-species differences have to be considered, these approaches have led to major breakthroughs and provided an increased potential to pre-clinical in vivo research [31,32].

Besides mimicking PCa phenotypes, GEMMs can also be used to study non-cancerous targets of hormone therapy. Multiple models studying the AR have been created from this perspective. Although these models do not directly result in novel prostate cancer treatments, their impact is indirect by improving the understanding of the AR and its pathways [34,35] (Table 2).

For example, in the TRAMP model, researchers altered the expression of SV40 antigens to create prostate cancer models reflecting the heterogeneous AR and p53 expression and castration response seen in patients [36].

### 2.3. Patient-Derived Models

Different patient-derived models have been reported, including primary cell cultures, patient-derived explants, and patient-derived xenografts [28]. These models are similar to other models, but are constructed from an actual tumor (Table 3). Consequently, these correlate better to the clinical setting than the pretreated cell lines.

At first, primary cells were grown in 2D cultures much like the cell lines discussed in Section 2.2, or they were grown in Matrigel to enable 3D culture. More recently, conditions have been optimized for 3D cultures (i.e., with printed scaffolds), spheroids, and organoids. As such, these primary cultures offer the possibility to expand tumor cells in vitro and even can be genetically modified. This enables their use in medium and high throughput assays for drug screening [28]. Although technically challenging, these models allow a detailed analysis in the study of the tumor microenvironment and cell–cell interactions [37]. Patient-derived organoids were successfully developed by Gao and colleagues in advanced disease stages [38]. More recently, these models have been shown to be very promising in reflecting rare tumor types (i.e., NEPC) and validating novel translational research methods (i.e., validation of single cell sequencing of prostate cancer) [39,40].

Patient-derived explants consist of primary tumor biopsies that are kept in their integrity in culture ex vivo. The culture conditions have been optimized considerably so that these models retain the functional and structural characteristics of the tissue, which enables their successful use in preclinical studies [41,42]. Consequently, these models create robust preclinical findings. Unfortunately, they cannot be propagated and will undergo changes after one or two weeks of culture, which limits their use to short term studies.

Patient-derived xenograft models are created by inserting tumor tissue in nude mice (see Section 2.1.2. These models allow timely interventions to be performed in living animals with a complete endocrine system. Downsides to xenografting are the lacking immune system, the gradual overgrowth of murine stromal cells, and the restriction to collect tissue only at the harvest time point [28].

### 2.4. The Ideal Preclinical Models

An ideal model for this setting would start from a heterogeneous untreated cell population of PCa. Models with a high throughput and methodologic flexibility are preferred to allow the study of different settings and interventions (Table 3). The available models are mostly derived from pre-treated metastatic patients. It is therefore challenging to find ideal preclinical models that faithfully reflect resistance to AR-targeted therapies. Most preclinical models are therefore only suitable to answer specific scientific questions. The lack of representative preclinical models undoubtedly explains the high failure rates of clinical trials with novel treatments [28].

## 3. Preclinical Models to Study AR Resistance Mechanisms

### 3.1. Reactivation of AR Signaling Output

#### 3.1.1. AR Overexpression

Androgen deprivation therapy blocks the production of gonadal androgens and subsequently lowers the androgen concentration in the circulation. PCa patients undergoing androgen deprivation often overexpress the AR by amplification of the AR gene [43]. This results in high AR activity despite the castrate levels of androgens in circulation. A large number of castration-resistant xenograft models display AR overexpression [44],⁠ suggesting AR overexpression to be sufficient to confer castration resistance.

Different cell lines have been developed to reflect resistance mechanisms to androgen deprivation and AR signaling inhibitors, including LNCaP-abl (androgen ablated), C4-2b, ResA/B, and 22Rv1 [7,11,45,46,47]. Interestingly, AR expression in these cell models is very heterogeneous: while some models have AR amplifications, others have unaltered or even reduced AR expression [7,11,48,49]. In addition, AR expression is not always correlated with response to AR targeting therapies. One should differentiate between cells that require AR activity for their growth and those that still express the AR but do not need it to maintain proliferation (AR indifferent) [50].

As the discussed preclinical models suggest that AR overexpression develops in settings with some remaining AR activity, more potent AR inhibition may prevent PCa cells from acquiring this mechanism of resistance. This could be achieved by highly potent anti-androgens (i.e., enzalutamide) that block AR activity even upon AR overexpression, and combination treatments [23]. Pre-clinical models with acquired resistance are best suited to investigate if these combination therapies lead to a change in the resistance mechanisms.

Another therapeutic possibility in AR-overexpressing tumors consists of supraphysiological androgen treatment. In PCa cell lines and PDX models, high testosterone concentrations inhibit the MYC-E2F pathway and induce DNA damage [27,51]. A recent phase II clinical trial confirmed that a subpopulation of patients with anti-androgen-resistant mCRPC showed radiographic responses to bipolar androgen therapy, and several larger clinical trials are currently ongoing [52].

#### 3.1.2. AR Mutation

Several mutations in the AR have been found in AR signaling inhibitors-resistant disease. Many of these mutations are located in the ligand binding pocket within the AR ligand binding domain. AR ligand binding domain mutations can either broaden the agonist spectrum (ligand promiscuity), prevent the binding of anti-androgens, or even convert anti-androgens into agonists [53,54]. This mechanism of resistance was first described in the LNCaP cells and later confirmed as a recurrent mutation in patient material. The T878A AR mutation, which was found in the LNCaP, confers ligand promiscuity and changes the earlier anti-androgen hydoxyflutamide into an agonist. This mutant remains sensitive to the second-generation AR signaling inhibitors. Other recurrent AR mutations found in disease resistant to the current AR antagonists are L702H, W742C, H875Y (also found in 22Rv1 cells) and F877L. The in-depth in vitro characterization of these mutations has identified underlying mechanisms, which are highly relevant for the treatment of patients which are positive for any of these mutations [55,56,57].

#### 3.1.3. AR Splice Variants

AR signaling inhibitor treatment has been shown to induce truncated versions of the AR in cell cultures as well as in patient material. The AR-V7 variant is most frequently expressed in mCRPC tissue and associated with a lower response to abiraterone and enzalutamide treatment [58]. Different cell lines, xenograft models, and GEMMs have described and studied different alternative splice variants in prostate cancer [59]. Alternative splicing and exon skipping has been described to generate such AR variants which lack the ligand binding domain. In the absence of the ligand binding domain, the AR is constitutively active. However, some studies in cell lines demonstrated that AR variants require the full-length receptor for their function, without the need for ligand activation [60].

Thus, drugs that target the transcription activation function in the amino terminal domain of the AR might provide a new therapeutic strategy. Such compounds are in the preclinical phase [61]. Other ways to overcome AR-V7 expression might be to target proteins that interact with it. For instance, AKR1C3 (an intratumoral enzyme converting dehydroepiandrosterone (DHEA) into testosterone and DHT) stabilizes ARV7 and can be targeted to reduce ARV7 levels and resensitize the cells to anti-androgens [47,62]. Interestingly, some AKR1C3 inhibitors have been shown to also bind to the DNA binding domain of the AR, and thus directly inhibit full length and variant AR activity [63].

#### 3.1.4. Glucocorticoid Receptor Takeover

The first evidence of glucocorticoid receptor (*NR3C1* or GR) and AR correlates was found upon proteasome inhibition, where there was an accumulation of GR with a lowering of AR [64]. The landmark paper of Arora et al. thereafter proved that the GR allows prostate cancer cells to bypass AR inhibition [65]. They and others demonstrated that the GR is frequently overexpressed in patient samples, LNCaP/AR xenograft tumors, as well as LNCaP-abl and DuCaP cells that have relapsed on second generation anti-androgens [65,66].

Interestingly, the GR contains a negative AR response element in its promoter that represses GR expression in cells with active AR signaling and thus provides a mechanistic explanation for GR upregulation in patients receiving AR-targeted therapies [67]. Importantly, the cistromes of the AR and GR are overlapping and a large number of transcriptional target genes are regulated by both transcription factors [65,68]. This is likely due to the high sequence conservation of the DNA binding domain in both receptors [69]. Therefore, the GR can bypass AR inhibition in prostate cancer cells by reactivating the downstream AR signaling output.

This might have serious implications in patients receiving abiraterone acetate, since this drug has to be given together with the glucocorticoid prednisone to mitigate side effects [70]. A post hoc analysis of the COU-AA-301 study found that the overall survival in patients treated with abiraterone and prednisone was shorter in those patients that already received glucocorticoids at baseline, but multivariate analysis could not confirm an independent prognostic effect [71]. A confounding factor could be that certain AR mutations lead to ligand promiscuity and allow glucocorticoids to directly activate the AR [72]. In addition, glucocorticoids are known to exert anti-tumorigenic effects in AR-positive and AR-negative prostate cancer cells [73,74]. Several clinical trials have tested the effect of glucocorticoids in mCRPC patients and observed favorable outcomes on quality of life and serum PSA levels [75].

In conclusion, glucocorticoids are an important part in the treatment of mCRPC patients, and systemic GR inhibition is therefore most likely not clinically applicable. However, as the preclinical models have demonstrated, the use of highly potent AR targeted therapies can turn the GR into a mediator of therapy resistance. Therefore, novel therapeutic approaches that target GR takeover of specific growth signaling might be beneficial for patients with reappearing GR in the tumor cells.

#### 3.1.5. AR Coactivators

The PCa cell lines have heterogenous AR activity which is mediated by co-activators. Recently, established LNCaP cells with high AR activity (but normal AR expression) showed reduced sensitivity to enzalutamide [76]. This was mediated by the AR coactivator GREB1. NCOA1 is a transcriptional co-regulator of several nuclear receptors and overexpressed in metastatic PCa [77]. NCOA1 knock-down reduced AR activity, proliferation, and migration [78]. Targeting NCOA1 is particularly interesting due to its importance for both the AR and the GR, which might target PCa cells with GR takeover. Another well-known coactivator is the p300/CBP complex, for which a specific inhibitor has been developed [79]. This inhibitor inhibits similar to enzalutamide histone acetylation occupancy at the PSA promotor and alters Myc expression in 22Rv1 cells and xenograft models [79]. Although studying these coactivators revealed their relevance in AR signaling, the specific value of these coactivators in AR signaling inhibitor resistance remains to be investigated.

#### 3.1.6. AR Outlaw Activation by Cytokines and Growth Factors

A recent study in mCRPC patients found IL6 levels to be associated with de novo abiraterone and enzalutamide resistance [80]. Cytokines and other growth factors were initially described as ligand-independent, but newer results indicate that it amplified the androgen dependent signal [42,81]. While this effect is best described for the pro-inflammatory cytokine IL6, other cytokines (LIF, IL8, IFNγ), endotoxins (LPS), growth hormones (IGF-I), and neuropeptides (bombesin, neurotensin) are also known to affect AR activity [42,82,83,84,85].

IL6 is a well-established regulator of PCa progression and circulating IL6 levels are a predictive biomarker for poor prognosis [86,87]. In LNCaP cells, IL6 over-expression and constitutive activation of the downstream mediator STAT3 block the effect of enzalutamide on proliferation, probably via enabling AR DNA-binding [88]. However, clinical trials targeting IL6 have failed [89,90], which led to a shift in research focus on other parts of the IL6 signaling cascade. STAT3 is the main downstream mediator of IL6 and is responsible for IL6-mediated AR outlaw activity [42,91]. Activated STAT3 is found in 95% of CRPC metastases [92], and STAT3 activation is increased upon enzalutamide treatment in LNCaP cells [88,93]. Small molecule inhibitors of STAT3, such as niclosamide and galiellalactone, were able to reduce AR outlaw activity and inhibit the proliferation of enzalutamide resistant cell lines⁠ [94,95].

Many unsolved questions remain. For instance, there is conflicting evidence with regards to the exact mechanisms of AR outlaw activation. In addition, there is no information available if outlaw activation alters the cistrome of the AR and thus changes the growth signals in the cells. This is further complicated by the fact that most pre-clinical research on this topic is limited to the LNCaP cell line, and it is thus difficult to generalize the findings. For instance, an ex vivo study with prostate tissue cultures of treatment-naive patients undergoing radical prostatectomies found that galiellalactone consistently reduces AR activity in benign tissue but has mixed effects in cancerous tissue [42]. Further preclinical and clinical research needs to define subpopulations that might benefit from therapies targeting AR outlaw activation.

### 3.2. Mechanisms That Bypass AR Signaling

#### 3.2.1. Neuroendocrine Transdifferentiation

Neuroendocrine prostate cancer (NEPC) is an aggressive, AR-negative form of PCa associated with poor prognosis for the patient. However, NEPC is an umbrella term that actually describes two very different kinds of cancer: (1) neuroendocrine carcinoma that developed within the prostate, or (2) prostate adenocarcinoma cells that transdifferentiated into a neuroendocrine-like PCa cells [96,97]. These forms differ greatly from each other, which resulted in an updated pathological classification [96,97,98].

Etiologically, GEMMs with a prostate-specific knock out of p53 and RB1 showed more neuroendocrine transdifferentiation, suggesting that this process is caused by cumulative mutations [99]. This sequential accrual of mutations was confirmed in recent xenografts of human circulating tumor cells [100]. Cell culture experiments subsequently demonstrated that stimulators of this transdifferentiation include MUC1-C [101] and the ERK/MAPK pathway [96,102].

#### 3.2.2. Stemness/Stem Cells

Stem cells and stem-like signatures reflect a regenerative and aggressive PCa phenotype. These tumors are characterized by specific surface markers (i.e., CD44^+^/alpha2beta1hi/CD133^+^) [103]. There is much debate on the development of these cell types, as stemness can pre-exist as well as occur via de-/trans-differentiation [104,105].

Stemness signatures are commonly associated with low AR signaling. Commercial cell lines PC 3 and DU 145 cells are AR-negative and express stemness markers [106,107]. The PSA−/lo (expressing low amounts of PSA) cell lines are characterized by stem cell signatures, self-renewal potential and treatment resistance [108].

Stemness markers moreover increase in hormone treated models. After androgen deprivation, increased stemness was seen in LuCaP35 prostate tumor explants [109]. Upon enzalutamide resistance, cell models showed increased stem cells and stemness markers [110].

## 4. Challenges and Therapeutic Opportunities

### 4.1. The Problem of Cross-Resistance

#### 4.1.1. To AR-Targeting Therapies

Since several AR-targeted therapies (e.g., cyproterone, flutamide, bicalutamide, enzalutamide, and synthesis inhibitor abiraterone) have been introduced in the clinic, it is crucial to know whether there is cross-resistance or whether there is an optimal sequence of treatments [2].

Hereby, preclinical studies were crucial to support the development of clinical trials. In cell lines, cross-resistance between AR-targeted therapies is commonly observed [11,46]. Sequential treatments with AR-targeted therapies have proven inferior to second line chemotherapeutics [111]. This is logic as the ultimate target of the compounds is the ligand binding pocket of the AR [11]. Added to the cross resistance between AR inhibitors, abiraterone resistant cells have lower responses to enzalutamide [112]. Possible mechanisms of cross-resistance have been suggested based upon resistant cell lines, including the AKR1C3/AR-V7 axis [46].

In conclusion, preclinical models show a strong cross resistance to AR targeting therapies. Clinical trials confirm the response to androgen deprivation treatment to be associated to the response to the new AR-targeting therapies, strengthening this hypothesis [113].

#### 4.1.2. To Chemotherapeutics

Cell culture experiments showed a clear cross resistance to the taxanes docetaxel and cabazitaxel in hormone refractory cell lines [111,112]. Xenograft models later explored the response of enzalutamide resistant PCa to taxane chemotherapeutics [114]. While there was clear cross resistance to docetaxel, cabazitaxel remained effective in these models. This could possibly indicate that cabazitaxel is more AR-independent in its action (with higher potency and microtubular inhibition) [114]. These findings are in line with retrospective clinical evidence confirming worse clinical outcomes of docetaxel after abiraterone treatment compared to untreated PCa patients [115,116]. Cabazitaxel retains its activity, regardless of previous abiraterone use [117,118,119].

### 4.2. Heterogeneity

As prostate cancer is a heterogeneous disease, clinical samples should be analyzed in depth to yield strong and highly specific markers that predict treatment response [40,120,121]. These translational studies should be supplemented with preclinical studies investigating the underlying biology by replicating disease states or investigating specific alterations.

For example, in Karthaus et al., the regenerative potential of prostate cells was studied at the single cell level at different time points after castration. By doing so, they investigated the changes and regenerative potential of murine and human cells [120,122].

### 4.3. Targeted Therapies

Novel targeted therapies are being developed to treat patients based on their individual molecular profile [123]. By revealing the underlying biology and treatment responses, preclinical models can improve the patient selection for these treatments and contribute to the development of novel treatments [123].

As an example, the AR overexpressing LNCaP cell line contributed to the development of AR signaling inhibitors [23].

Although the value of novel treatments is determined in clinical trials, a tight association between preclinical treatment response and the clinical response of PCa patients is seen [124]. Novel therapies can be screened rapidly using the preclinical responses in high throughput models [125]. Good examples of this are the 110 tested drug panel on CRPC LuCaP PDX organoid and tissue cultures in PCa [124,125,126,127]. This is supplemented by PRISM, a large repurposing screen, testing 4518 compounds against more than 570 cell lines [128].

Preclinical models are a fundamental aspect in prostate cancer research and often start as proof of principle (cell lines), to be replicated in animal models and finally in clinical trials. As translational studies unravel novel possibilities for targeted therapy, preclinical models will be essential in the development of these therapies.

## 5. Conclusions

Basic and translational insights in the molecular mechanisms of androgen synthesis and AR action all started from cell lines, together with biophysical studies of the AR (like DNA and ligand binding) and, more recently, complex formation with coregulators [4]. The AR-targeted therapies that are based on this knowledge have undergone validation in different preclinical models of PCa. Although the preclinical models have many disadvantages, major advances in the field of PCa depend on the application of preclinical models.

Preclinical models will prove to be important in assessing what therapies are likely to be effective based on the patient profile. Before we will be able to run clinical trials with drugs likely to be effective, thorough insights in the underlying prostate cancer biology and treatment resistance are needed.

## Figures and Tables

**Table 1:** Overview commonly used prostate cancer cell lines. Wild type (WT); androgen receptor (AR). Adapted from [6,7,8].

Cell Line	AR/PSA	Hormone Resistant/Sensitive	Direct/Xenograft (Indirect)	Specific Characteristics
**Lymph node metastasis**				
LNCaP	+	Sensitive	Direct	T877A AR mutant, PTEN -, androgen dependent
LAPC-4	+	Sensitive	Xenograft	P53 mutant, WT PTEN
**Bone metastasis**				
VCaP	+	Sensitive	Xenograft	Amplified WT AR, AR splice variants, TMPRSS-ERG fusion
MDA PCa 2a/b	+	Sensitive	Direct	AR mutants L702H and T878A
PC3	−	Resistant	Direct	
CWR22(Rv1)	+	Resistant	Xenograft	H875Y AR mutant, AR splice variants, WT PTEN, Zinc Finger Duplication
**Organ metastasis**				
DU-145 (brain)	−	Resistant	Direct	
DuCaP (dura)	+	Sensitive	Xenograft	Amplified WT AR

**Table 2:** Overview of possible targetable pathways for genetically engineered mouse models (GEMM) of prostate cancer (PCa). Adapted from [32].

GEMM	PCa Inducement	Specific Characteristics
**Localized PCa alterations**		
TMPRSS-ERG fusion	+ −	Modest phenotype on its own, additive effect with PTEN loss
SPOP mutation	+ −	Modest phenotype on its own, additive effect with PTEN loss
**Advanced PCa alterations**		
PTEN loss	+	Most frequent GEMM, Clinical stage ranges between models. Additive effects with other alterations
Tumor surpressor genes losses (RB/p53)	+ −	Modest phenotype on its own, additive effect with PTEN/BRCA mutations/RB/p53 losses
DNA repair genes mutation	+	Fewer models available, study of PARP inhibitors. Additive effects with PTEN/RB/p53 losses
Oncogenes	+	Clinical stage ranges between Myc levels, additive effects with p53, and PTEN models
**Other models—mechanistic studies of targets**		

**Table 3:** Overview of the advantages and disadvantages of different preclinical models. General PCa-derived and patient-derived models (indicated in color) are discussed. Prostate cancer, PCa.

Model		Advantages/Disadvantages
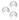	PCa cell line experiments	+ Easy to culture, high-throughput system − Very homogeneous, not reflective of clinical tumors
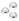	Genetically modified PCa cell lines	+ Easy validation of de novo resistance mechanisms and alterations from databases − Acquired resistance more difficult to study
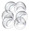	3D cell cultures/organoids	+ Study of Cell-Cell interaction − Scaffold material can alter cellular processes
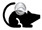	Mouse Xenografts of PCa	+ Study of metastasis and treatment response in vivo + Working endocrine system − Limitations similar to injected cell lines
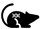	Genetically modified mouse models	+ In vivo validation of genetic alterations and underlying pathways − Inter species differences, questionable generalizability to human prostate cancers
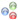	Patient derived cell lines	+ Good correlation with specific human tumor + Reproducibility of experiments, high throughput − Only representing a certain tumor type, no cell-cell interaction
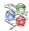	Patient derived tissue cultures	+ Excellent representation of respective type of prostate cancer − Limited life span, cannot be propagated
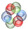	Patient derived 3D cell cultures/organoids	+ Study of Cell-Cell interaction in specific tumor subtypes + Medium to High throughput − Scaffold material can alter cellular processes − Technically challenging
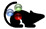	Patient derived Xenografts	+ Study of metastasis and treatment of a certain tumor in vivo + Working endocrine system − No immune system, overgrowing murine tissue, tissue collection only at endpoint.
